# Retraction: Stiffening-Induced High Pulsatility Flow Activates Endothelial Inflammation via a TLR2/NF-κB Pathway

**DOI:** 10.1371/journal.pone.0220600

**Published:** 2019-07-29

**Authors:** Yan Tan, Pi-Ou Tseng, Daren Wang, Hui Zhang, Kendall Hunter, Jean Hertzberg, Kurt R. Stenmark, Wei Tan, The PLOS ONE Editors

After publication of this article [1], concerns were raised about similarities between the following bands in the western blot panels shown in Figs 2, 3, and 4:

- Fig 2B MCP-1 lanes 1, 2 and Fig 3D (left panel) MCP-1 lanes 1, 2
- Fig 2B MCP-1 lane 3 and Fig 3B MCP-1 lane 4
- Fig 2B GAPDH lane 1, Fig 3B GAPDH lane 5, and Fig 3D (left panel) GAPDH lane 4
- Fig 2B GAPDH lane 2, Fig 3B GAPDH lane 2, Fig 3D (left panel) GAPDH lane 2, and Fig 4C GAPDH lane 1
- Fig 3B GAPDH lane 3 and Fig 4C GAPDH lane 2
- Fig 3B GAPDH lane 4, Fig 3D (left panel) GAPDH lane 3, and Fig 4C GAPDH lane 4
- Fig 3B, GAPDH lane 6, Fig 3D (left panel) GAPDH lane 1, and Fig 4C GAPDH lane 3

The authors provided some replication data in support of the results, but the original uncropped images underlying the published figures are no longer available.

In light of these unresolved concerns, the authors and *PLOS ONE* Editors retract this article.

All authors agreed with the retraction.
