# Correction: Retraction: Stiffening-Induced High Pulsatility Flow Activates Endothelial Inflammation via a TLR2/NF-κB Pathway

**DOI:** 10.1371/journal.pone.0221184

**Published:** 2019-08-08

**Authors:** The PLOS ONE Staff

## Notice of republication

The following sentence was not included correctly in the original Notice of Retraction: “The authors provided some replication data in support of the results, but the original uncropped images underlying the published figures are no longer available.”

The publisher apologizes for the error. The Notice of Retraction was republished on August 1, 2019 to correct this error. Please download the notice again to view the correct version.
