# Correction: Retraction: Stiffening-Induced High Pulsatility Flow Activates Endothelial Inflammation via a TLR2/NF-κB Pathway

**DOI:** 10.1371/journal.pone.0224014

**Published:** 2019-10-10

**Authors:** The PLOS ONE Staff

## Notice of Republication

The byline in the original Notice of Retraction [1] was incorrect. The publisher apologizes for this error. The Notice of Retraction was republished on October 1, 2019 to correct for this error. Please download the retraction notice [1] again to view the correct version. The retracted article [2] can be viewed at https://doi.org/10.1371/journal.pone.0102195.
